# Supplementary material for: Exploring gender dysphoria and related outcomes in a prospective cohort study: protocol for the Swedish Gender Dysphoria Study (SKDS)
Source: BMJ Open. 2023 Apr 19;13(4):e066571. doi: 10.1136/bmjopen-2022-066571 (PMC10124297; doi:10.1136/bmjopen-2022-066571)
Supplement: Author's manuscript [file bmjopen-2022-066571.draft_revisions.pdf]

BMJ Open

BMJ Open is committed to open peer review. As part of this commitment we make the peer review history of every article we publish publicly available.

When an article is published we post the peer reviewers' comments and the authors' responses online. We also post the versions of the paper that were used during peer review. These are the versions that the peer review comments apply to.

The versions of the paper that follow are the versions that were submitted during the peer review process. They are not the versions of record or the final published versions. They should not be cited or distributed as the published version of this manuscript.

BMJ Open is an open access journal and the full, final, typeset and author-corrected version of record of the manuscript is available on our site with no access controls, subscription charges or pay-per-view fees (http://bmjopen.bmj.com).

If you have any questions on BMJ Open's open peer review process please email info.bmjopen@bmj.com

BMJ Open

Exploring gender dysphoria and related outcomes in a prospective longitudinal cohort: Swedish Gender Dysphoria Study (SKDS)

Journal:	*BMJ Open*
Manuscript ID	bmjopen-2022-066571
Article Type:	Protocol
Date Submitted by the Author:	09-Aug-2022

Complete List of Authors:	Özel, Fatih; Uppsala University, Department of Organismal Biology; Uppsala University, Centre for Women's Mental Health during the Reproductive Lifespan – WOMHER<br>Indremo, Malin; Uppsala University, Department of Medical Sciences, Psychiatry<br>Karamanis, Georgios; Uppsala University, Department of Medical Sciences, Psychiatry<br>Elofsson, Ulf; Uppsala University, Department of Medical Sciences, Psychiatry; Uppsala University, Department of Women's and Children's Health<br>Beckman, Ulrika; Sahlgrenska University Hospital, Department of Psychiatry<br>Fazekas, Attila; Lund University, Department of Psychiatry<br>Frisén, Louise; Karolinska Institute, Department of Clinical Neuroscience<br>Isaksson, Magnus; Uppsala University, Department of Medical Sciences<br>Sandström, Lotta; Karolinska University Hospital, ANOVA; Umeå Universitet, Department of Clinical Sciences, Psychiatry<br>Thelin, Nils; Linköping University Hospital, Division of Psychiatry<br>Tivesten, Åsa; University of Gothenburg, Wallenberg Laboratory for Cardiovascular and Metabolic Research, Department of Molecular and Clinical Medicine, Institute of Medicine, Sahlgrenska Academy; Sahlgrenska University Hospital, Department of Endocrinology<br>Wahlberg, Jeanette; Örebro University, Faculty of Medical Sciences<br>Skalkidou, Alkistis; Uppsala University, Department of Women's and Children's Health<br>Bodlund, Owe; Umeå Universitet, Department of Clinical Sciences, Psychiatry<br>Papadopoulos, Fotios C.; Uppsala University, Department of Medical Sciences, Psychiatry

Keywords:	Child & adolescent psychiatry < PSYCHIATRY, Adult psychiatry < PSYCHIATRY, EPIDEMIOLOGY

SCHOLARONE™ Manuscripts

Exploring gender dysphoria and related outcomes in a prospective longitudinal cohort: Swedish Gender Dysphoria Study (SKDS)

Fatih Özel[1,2], Malin Indremo[3], Georgios Karamanis[3], Ulf Elofsson[3,4], Ulrika Beckman[5], Attila Fazekas[6], Louise Frisen[7], Magnus Isaksson[3], Lotta Sandström[8,9], Nils Thelin[10], Åsa Tivesten[11,12], Jeanette Walhberg[13], Alkistis Skalkidou[4], Owe Bodlund[9], Fotios C Papadopoulos[3]

[1]Department of Organismal Biology, Uppsala University, Uppsala, Sweden
[2]Centre for Women's Mental Health during the Reproductive Lifespan – WOMHER, Uppsala University, Uppsala, Sweden
[3]Department of Medical Sciences, Uppsala University, Uppsala, Sweden
[4]Department of Women's and Children's Health, Uppsala University, Uppsala, Sweden
[5]Department of Psychiatry, Sahlgrenska University Hospital, Region Västra Götaland, Gothenburg, Sweden
[6]Department of Psychiatry, Lund University, Lund, Sweden
[7]Department of Clinical Neuroscience, Karolinska Institutet, Stockholm, Sweden
[8]ANOVA, Karolinska University Hospital, Stockholm, Sweden
[9]Department of Clinical Sciences/Psychiatry, Umeå University, Umeå, Sweden
[10]Division of Psychiatry, Linköping University Hospital, Linköping, Sweden
[11]Wallenberg Laboratory for Cardiovascular and Metabolic Research, Department of Molecular and Clinical Medicine, Institute of Medicine, Sahlgrenska Academy, University of Gothenburg, Gothenburg, Sweden
[12]Department of Endocrinology, Sahlgrenska University Hospital, Region Västra Götaland, Gothenburg, Sweden
[13]Faculty of Medical Sciences, Örebro University, Örebro, Sweden

Correspondence to:
Fotios C Papadopoulos
fotis.papadopoulos@neuro.uu.se

For peer review only

Abstract

Introduction

There has been a drastic increase in the reported number of people seeking for gender dysphoria in many countries over the last two decades. Yet, our knowledge of gender dysphoria and related outcomes is restricted due to the lack of high-quality studies employing comprehensive approaches. This longitudinal study aims to enhance our knowledge of gender dysphoria; different aspects will be scrutinized, focusing primarily on the psychosocial and mental health outcomes, prognostic markers, and secondarily on the underlying mechanisms for its origin.

Methods and analysis

The Swedish Gender Dysphoria Study is an ongoing multicenter longitudinal cohort study with 501 registered participants with gender dysphoria who are 15 years old or older. Participants at different phases of their clinical evaluation process can enter the study, and the expected follow-up duration is 3 years. The study also includes a comparison group of 458 age and county matched individuals without gender dysphoria. Data on the core outcomes of the study, which are gender incongruence and experienced gender dysphoria, body satisfaction, and satisfaction with gender-affirming treatments, as well as other relevant outcomes including mental health, social functioning and life satisfaction are collected via web surveys. Two different research visits, before and after starting on gender-affirming hormonal treatment (if applicable), are planned to collect respective biological and cognitive measures. Data analysis will be performed using appropriate biostatistical methods. A power analysis showed that the current sample size is big enough to analyze continuous and categorical outcomes, and participant recruitment will continue until December 2022.

Ethics and dissemination

The ethical permission for this study was obtained from the Local Ethical Review Board in Uppsala, Sweden. Results of the study will be presented at national and international conferences and published in peer-reviewed journals. Dissemination will also be implemented through the Swedish Gender Dysphoria Study network in Sweden.

Strengths and limitations of this study

- The longitudinal design with a large sample size is the biggest strength of this multicenter cohort study, considering that gender dysphoria is an uncommon diagnosis.
- Having a comparison group consisting of individuals without gender dysphoria stands as a valuable advantage.
- A comprehensive assessment of psychosocial determinants is being performed.
- Participants enter the study at different stages of their clinical evaluation process, which partly results in missing baseline information.
- The representativeness of the target population could be affected by the fact that individuals with gender dysphoria who have more mental health issues tend to participate less often.

For peer review only

Introduction

Gender dysphoria (GD) is, according to the latest version of the Diagnostic and Statistical Manual of Mental Disorders (DSM-5),[1] a condition that involves distress due to incongruence between an individual's birth-assigned sex and gender identity. During the last decades, there has been an increasing number of individuals with GD seeking gender-affirming health care in many countries.[2 3] This increase is more pronounced among the younger age groups, and is more noticeable among individuals who are assigned female at birth (AFAB).[4] Similar rising patterns for GD have also been demonstrated in Sweden recently. An increase in the incidence of GD was observed during the period 2004-2015 in Sweden, with the most profound increase among AFAB in the age group 10-17 (approximately 20-30% average yearly increase), while 10-17 year old assigned male at birth (AMAB) and both AFAB and AMAB in the age group 18-30 years had an average yearly increase of approximately 10-20%.[5] In the latest report of the National Board of Health and Welfare, 0.06% of the Swedish population was stated to have a GD diagnosis in 2018.[6] Even though an increasing trend in GD and the consequent need for gender-affirming health care is widely recognized, our understanding of GD and gender-affirming health care is substantially limited in many aspects.

The origin of GD is unknown but assumably multifactorial, with biological factors interacting with the psychosocial environment. There are established sex differences in the brain, concerning neuroanatomy, neurochemistry, as well as in neurocognition.[7] One hypothesis for the emergence of GD is that the processes of sexual differentiation of the genitals and the brain may be incongruent.[7] However, evidence from multiple neuroimaging studies has not given support to this. An alternative hypothesis that is informed by studies finding similar patterns in individuals with GD, and which differ from cisgender men and women, is that an important mechanism behind gender incongruence and often associated distress (GD) is a disconnection between cerebral circuits mediating perception of self and those mediating perception of own body.[8 9] Genetic factors have also been observed to play a role in GD, in part exerting their effects through altered sex hormone signaling; the estrogen and androgen receptors seem to be implicated in the genetic basis of GD.[10] Moreover, epigenetic alterations among individuals with GD have been recently proposed,[11] supporting evidence highlighting the importance of the interaction between genetic architecture and environment in the development of GD. Yet, the current evidence on biological determinants of GD are inconclusive in the literature.

An important aspect in the field of GD research relates to the mental health of individuals with GD. A growing body of evidence shows that persons with GD are more vulnerable to mental illnesses. A recent study utilizing Swedish population register data has reported that mood and anxiety disorders, use of antidepressant or anxiolytic medication and hospitalization rates due to suicide attempts are more common among persons with GD in comparison to the general population.[12] In addition to psychiatric diagnoses, transgender persons' quality of life is radically affected, with reported lower life satisfaction.[13] The aforementioned adverse consequences on the mental well-being of individuals with

For peer review only

GD could be explained through stigma-related stress. This framework is conventionally called minority stress and was established almost 20 years ago.[14] According to the minority stress model, gender minority groups endure particular stressors such as discrimination, lack of social support and violence and this intense exposure to stressors could result in mental health issues. Individuals with GD are unfortunately no exception to minority gender groups and their health is influenced by different sources of stigma-related stress.[13 15] More research is required on the experience of discrimination and violence by persons with GD and its effects on their mental health.

Gender-affirming medical treatment is an additional dimension that needs to be addressed in research. Treatment guidelines are provided in the World Professional Association for Transgender Health's (WPATH) Standards of Care, currently in its 7th version.[16] Gender-affirming hormonal treatment (GAHT) consists mainly of estrogens and antiandrogens for AMAB and testosterone for AFAB. The goal of GAHT is to induce the development of bodily characteristics congruent with the self-identified gender, thereby improving mental health and quality of life. For children and adolescents, puberty suppression with GnRH analogues is implemented to delay the development of secondary sexual characteristics. The intention is to alleviate the distress associated with the development of secondary sex characteristics, thereby providing a time for ongoing discussion and exploration of gender identity before deciding whether to take more irreversible steps. Previous studies posit that GAHT ameliorates the symptoms of GD, increases the quality of life, alleviates depression, anxiety, suicidality and improves psychosocial functioning among both young and adult individuals.[17-20] One should still acknowledge the limitations of interpreting the results from previous research. Moreover, the reports on the potential adverse health outcomes related to GAHT make research in this dimension even more indispensable.[21 22]

There has been an increase in research studies related to GD in parallel to the increase in GD incidence. Still, we lack evidence focused on some particular aspects of GD; such as biological and psychological determinants, mental health and psychosocial outcomes and the impact of gender-affirming treatment (GAT) on these outcomes. The majority of the studies conducted in the field of GD have methodological limitations, with small sample sizes, cross-sectional design and no comparison groups. Larger longitudinal studies are required to improve our understanding of GD and associated outcomes. More research is especially warranted among adolescents and young adults with GD, as those are the age groups with the most profound incidence increase during the last decade.

The Swedish Gender Dysphoria Study (Svenska Könsdysforistudien - SKDS) thus aims to investigate (1) mental health outcomes associated with GD, for instance, depressive and anxiety symptoms and suicidality; (2) psychosocial outcomes in relation to GD, such as quality of life, global functioning and social vulnerability (along with support, discrimination and violence); (3) biological correlates of GD containing the effects of early-life environmental exposures, genetic underpinnings, epigenetic alterations and potential prognostic biological markers; (4) different aspects of GAT, including the impact on mental health and psychosocial outcomes.

For peer review only

Methods and analysis

Study design and participants

SKDS (www.skds.se) is an ongoing multicenter longitudinal cohort study in Sweden, which was established in 2016. All participants in contact with one of the associated gender dysphoria evaluation clinics are eligible to be asked to participate. Having contact with a clinic for gender dysphoria, being at least 15 years old and speaking Swedish were set as the inclusion criteria. Relevant information about the study is sent to all participants and written informed consent is obtained. The right of leaving the study without any obligation to report a reason for that and the fact that participating or not in the study has no effect on the received healthcare is clearly indicated during the informed consent process. Separate informed consents are asked for different parts of the study (e.g. participating in surveys, research visits, online cognitive tests, giving biological samples, etc). Recruitment of participants is ongoing in the GD clinics in Alingsås/Gothenburg, Linköping, Lund/Malmö, Stockholm (center for evaluation of children), Umeå, and Uppsala. In Sweden, the evaluation process and treatment for GD is centralized to these six regional clinics working in multidisciplinary teams, all following a national consensus program.

The participants of SKDS are followed up longitudinally from inclusion to 3 years later, with several assessments of variables of interest. Due to the naturalistic design of the study, the participants can be at different phases in their clinical evaluation process when they enter the study. The recruitment of the participants is expected to be finished in December 2022; therefore, the follow-up is predicted to be finalized in December 2025.

There are currently 501 registered participants with GD in SKDS. In January 2019, the governmental agency Statistics Sweden provided 30 random controls with no registered diagnosis of GD up to that time (15 with male legal gender and 15 with female legal gender) for each participant in the study. To recruit controls, an invitation letter and one reminder were sent via mail to 8790 persons in total; a comparison group thus was constituted with 458 age and county matched controls without a diagnosis of GD. The controls are only assessed once with the same baseline instruments as the participants with GD. Regarding the low participation rate for the controls, the dropout analysis conducted by Statistics Sweden showed higher dropout rates for controls with male legal gender, those born outside Sweden and those without Swedish citizenship. No other major biases were observed for age, income or municipality. The second round of recruitment of controls will be executed at the beginning of 2023, in order to acquire more controls for recruited participants since 2019.

In order to plan and follow the study coordination and progress as well as the utilization of all collected data, a national steering committee for the SKDS was created in November 2015, consisting of clinicians and researchers in the field of psychiatry and endocrinology from the collaborating clinics. Representatives from interest organizations, including Transammans and RFSL Ungdom in Stockholm and Uppsala, have an advisory role in the SKDS.

Data collection and measures

For peer review only

1. Main outcomes and psychosocial measures

Longitudinal web surveys are the main tools for data collection in the current study. Sociodemographic characteristics and outcomes of relevance for GD are measured through previously validated instruments listed in Table 1, as well as three specific surveys for SKDS, Surveys I-III, described in Table 2. The core outcomes of the current study are gender incongruence and experienced gender dysphoria, body satisfaction, and satisfaction with GAT. Assessment of the satisfaction with each of the received GAT, as well as overall satisfaction with GAT, is covered in depth in the last SKDS-specific survey. For each of the received treatments, the participants are asked the following questions: ”how satisfied are you with the physical result of treatment x?”, ”how has treatment x affected your mental well-being?” and “if you were to decide on initiating treatment x today, would you make the same decision again?”.

In addition to the core outcomes, the included instruments cover general mental health status, previous and current psychiatric diagnoses, traits of autism spectrum disorder and attention deficit hyperactivity disorder, alcohol and substance use, social functioning and life satisfaction. Furthermore, a scale on potential side effects of treatment is administered after the initiation of GAT.

2. Cognitive measures

All participants who have not started on gender-affirming hormonal treatment are invited to undertake a computer-based neurocognitive assessment battery, called WebNeuro, which can be administered online or with a local installation.[23] Different domains are addressed within the battery including sensorimotor, memory, executive functioning, attention, and social cognition. Somatomaps, an online tool for assessing body perception, is also administered to all participants.[24]

Patients recruited from the gender dysphoria evaluation clinic in Uppsala are also invited to a research visit at the Uppsala University Hospital where they are asked to undertake specific neurocognitive tests called Cambridge Gambling Task (CGT) and Stockings of Cambridge (SOC) from the CANTAB battery.[25] All cognitive tests are performed at baseline, before gender-affirming hormonal treatment and at least six months after.

3. Biological measures

For genetic and epigenetic analyses, all participants are asked to contribute with saliva samples, deciduous teeth and the consent to use newborn dried blood spots which are stored in the national PKU-biobank. Saliva samples will also be collected after a period of at least six months after starting on GAHT for individuals without GAHT at baseline in order to do epigenetic analyses related to the GAHT.

Participants recruited from the gender dysphoria evaluation clinic in Uppsala are also invited to take part in a research visit, contributing to more biological measures before and after the initiation of GAHT. The measures consist of blood samples to analyze hormonal levels and possible changes in the immune system, a physical examination including blood pressure, weight and hip-waist ratio, as well as

For peer review only

measuring bioimpedance and heart rate variability. The first research visit is conducted during baseline assessment and a follow-up visit at least six months after initiation of GAHT.

These biological samples will be used in order to study potential biological correlates of GD consisting of the effects of early-life environmental exposures, genetic underpinnings, epigenetic alterations as well as potential prognostic biomarkers.

4. Electronic Health Records (EHR) and national register data

Information related to participants' gender dysphoria evaluation can be retrieved from the EHR for those who have given their consent. Similarly, for those with consent, a linkage will be made to the Swedish national registers, such as National Patient Register at the National Board of Health and Welfare and Statistics Sweden. These two information sources will be used to supplement the information collected with the web surveys.

5. Qualitative interviews with relatives

Participants are also asked for consent to contact a relative in order to recruit for other studies that explore relatives' experiences of gender dysphoria evaluation processes in health care.

Statistical Analysis

The research team of SKDS will perform the statistical analyses using the most recent version of the statistical software R. We will manage different types of missing data with different approaches. First, a potential bias that could arise from loss to follow-up will be evaluated; characteristics of missing participants will be addressed with dropout analysis and presented. Second, missing data and loss to follow-up will be diminished with chart review of clinical records and linkage to national health registers. We will also execute multiple imputation where it is required, and pooled results will be presented from imputed datasets.

Initially, summary statistics including the descriptive characteristics of participants will be provided. Cross-sectional and longitudinal relationships will be assessed using the distinctive outcomes we mentioned above. Logistic regression analysis will be used for the categorical outcomes, such as having been diagnosed with GD or not. Continuous outcomes will be analyzed with linear regression analysis. For longitudinal relations, we will conduct linear mixed models with repeated measures. We will use directed acyclic graphs (DAG) to identify confounders and mediators. Confounders will be adjusted in different models, whereas potential mediators will also be assessed with mediation analysis. We will apply multiple testing correction wherever necessary.

Regarding statistical power analysis, we need data for 52 participants of each birth-assigned sex in order to detect a 25% increase from a mean TCS-score of 2.1, with alpha=0.05 and power=90%, using a Mann-Whitney U test for the outcome of GD. Concerning the categorical mental health outcomes, expecting a change from 30% to 15% (as we have seen for the prevalence of self-harm thoughts in preliminary analysis from the SKDS-data), we need 161 participants for alpha=0.05 and power=90%, using a chi-squared test. For a smaller change from 30% to 20%, we need 294 participants for

For peer review only - http://bmjopen.bmj.com/site/about/guidelines.xhtml

alpha=0.05 and power=80%. The participation rate for SKDS is approximately 70%. Among those participants, approximately 70% actively participate in the majority of our surveys. The expected final number of recruited participants in the study is around 600, which leaves nearly 400 actively engaged participants.

Patient and public involvement

The study was originally designed by the research group comprising of clinicians and researchers working clinically with patients with GD. During the recruitment phase a collaboration was established with representatives from interest organizations; this collaboration is ongoing with regular meetings. The researchers were informed by the representatives concerning the contemporary needs of individuals with GD, and the research questions, methods and outcome measures were adjusted and further developed based on the feedback received. Interest organizations will be asked to contribute to the dissemination of the results through their networks. The results will also be disseminated through the project's website which is publicly accessible.

Ethics and dissemination

Ethical awareness has critical importance in clinical studies since patients might feel pressured to participate to access health care services. In SKDS, the voluntary participation and the fact that participation has no effect whatsoever on received care are clarified both orally and in writing. Furthermore, data breach might become an important risk, as sensitive information at the individual level is stored. However, we have taken all measures, such as pseudonymization, storage of the data on secure servers at Uppsala University and separating the personal identifiers (i.e. name and personal identity number) from the self-reports and other collected data. The study was approved by the Ethical Review Board in Uppsala (Dnr:2016/013).

We plan to disseminate the research findings from the SKDS through publication in peer-reviewed journals and presentation at relevant national and international conferences, as well as at dedicated seminars or workshops organized by the involved researchers, which could be also of interest to governmental and non-governmental bodies. Dissemination will be assured through the project's website (www.skds.se), as well as social media platforms. The results will be delivered to all clinical teams working with GD evaluation in Sweden through direct communication within the SKDS network. Contact will also be initiated with the national societies of general physicians, psychiatrists, psychologists, endocrinologists, as well as obstetricians and gynaecologists in Sweden.

Authors' contributions

AF, AS, JW, NT, OB, UB and ÅT were responsible for the conception and design of the study. FÖ drafted the protocol with significant contributions by MI, GK and UE. All authors have critically revised the draft for intellectual content and approved the final manuscript.

Funding statement

For peer review only

This work is supported by Region Uppsala, the Foundation of Nicke and Märta Nasvell, FORTE (dnr: 2021-01968) and Centre for Women's Mental Health during the Reproductive Lifespan – WOMHER.

Competing interests statement

None declared.

Acknowledgments

We would like to express our gratitude to all the study participants and the clinicians at the collaborating clinics. Moreover, we acknowledge Lucas Blixt from RFSL Ungdom Uppsala, Tom Summanen from Transammans and Vierge Hård from RFSL Ungdom for their advisory roles in the study.

References

1. American Psychiatric Association. Diagnostic and Statistical Manual of Mental Disorder 5th edition. *American Psychiatric Pub* 2013.
2. Arcelus J, Bouman WP, Van Den Noortgate W, *et al*. Systematic review and meta-analysis of prevalence studies in transsexualism. *Eur Psychiatry* 2015;30:807-815.
3. de Graaf NM, Carmichael P, Steensma TD, *et al*. Evidence for a Change in the Sex Ratio of Children Referred for Gender Dysphoria: Data From the Gender Identity Development Service in London (2000-2017). *J Sex Med* 2018;15:1381-1383.
4. Aitken M, Steensma TD, Blanchard R, *et al*. Evidence for an altered sex ratio in clinic-referred adolescents with gender dysphoria. *J Sex Med* 2015;12:756-763.
5. Indremo M, White R, Frisell T, *et al*. Validity of the Gender Dysphoria diagnosis and incidence trends in Sweden: a nationwide register study. *Sci Rep* 2021;11:16168.
6. Socialstyrelsen, Utvecklingen av diagnosen könsdysfori, 2020. Available: https://www.socialstyrelsen.se/globalassets/sharepoint-dokument/artikelkatalog/ovrigt/2020-2-6600.pdf
7. Smith ES, Junger J, Derntl B, *et al*. The transsexual brain--A review of findings on the neural basis of transsexualism. *Neurosci Biobehav Rev* 2015;59:251-266.
8. Manzouri A, Savic I. Possible Neurobiological Underpinnings of Homosexuality and Gender Dysphoria. *Cereb Cortex* 2019;29:2084-2101.
9. Majid DSA, Burke SM, Manzouri A, et al. Neural Systems for Own-body Processing Align with Gender Identity Rather Than Birth-assigned Sex. *Cereb Cortex* 2020;30:2897-2909.
10. Foreman M, Hare L, York K, *et al.* Genetic Link Between Gender Dysphoria and Sex Hormone Signaling. *J Clin Endocrinol Metab* 2019;104(2):390-396.
11. Ramirez K, Fernández R, Collet S, *et al.* Epigenetics Is Implicated in the Basis of Gender Incongruence: An Epigenome-Wide Association Analysis. *Front Neurosci* 2021;15:701017.
12. Bränström R, Pachankis JE. Reduction in Mental Health Treatment Utilization Among Transgender Individuals After Gender-Affirming Surgeries: A Total Population Study. *Am J Psychiatry* 2020;177:727-734.
13. Bränström R, Pachankis JE. Country-level structural stigma, identity concealment, and day-to-day discrimination as determinants of transgender people's life satisfaction. *Soc Psychiatry Psychiatr Epidemiol* 2021;56:1537-1545.
14. Meyer IH. Prejudice, social stress, and mental health in lesbian, gay, and bisexual populations: conceptual issues and research evidence. *Psychol Bull* 2003;129:674-697.
15. White Hughto JM, Reisner SL, Pachankis JE. Transgender stigma and health: A critical review of stigma determinants, mechanisms, and interventions. *Soc Sci Med* 2015;147:222-231.
16. Coleman E, Bockting W, Botzer M, *et al*. Standards of care for the health of transsexual, transgender, and gender-nonconforming people, version 7. *IntJ Transgend* 2012;13(4):165-232.
17. Murad MH, Elamin MB, Garcia MZ, *et al*. Hormonal therapy and sex reassignment: a systematic review and meta-analysis of quality of life and psychosocial outcomes. *Clin Endocrinol (Oxf)* 2010;72:214-231.

18. Mahfouda S, Moore JK, Siafarikas A, *et al*. Puberty suppression in transgender children and adolescents. *Lancet Diabetes Endocrinol* 2017;5:816-826.
19. Chew D, Anderson J, Williams K, *et al*. Hormonal Treatment in Young People With Gender Dysphoria: A Systematic Review. *Pediatrics* 2018;141:e20173742.
20. Kuper LE, Stewart S, Preston S, *et al*. Body Dissatisfaction and Mental Health Outcomes of Youth on Gender-Affirming Hormone Therapy. *Pediatrics* 2020;145:e20193006.
21. Getahun D, Nash R, Flanders WD, *et al.* Cross-sex Hormones and Acute Cardiovascular Events in Transgender Persons: A Cohort Study. *Ann Intern Med* 2018;169:205-213.
22. Nota NM, Wiepjes CM, de Blok CJM, *et al.* Occurrence of Acute Cardiovascular Events in Transgender Individuals Receiving Hormone Therapy. *Circulation* 2019;139:1461-1462.
23. Silverstein SM, Berten S, Olson P, *et al.* Development and validation of a World-Wide-Web-based neurocognitive assessment battery: WebNeuro. *Behav Res Methods* 2007;39:940-949.
24. Ralph-Nearman C, Arevian AC, Puhl M, *et al.* A Novel Mobile Tool (Somatomap) to Assess Body Image Perception Pilot Tested With Fashion Models and Nonmodels: Cross-Sectional Study. *JMIR Ment Health* 2019;6:e14115.
25. Available: https://www.cambridgecognition.com/products/cognitive-research/
26. Iliadis SI, Axfors C, Friberg A, *et al.* Psychometric properties and concurrent validity of the Transgender Congruence Scale (TCS) in the Swedish setting. *Sci Rep* 2020;10:18701.
27. Lindgren TW, Pauly IB. A body image scale for evaluating transsexuals. *Arch Sex Behav* 1975;4:639-656.
28. Zucker K, Mitchell J, Bradley S, *et al.* The Recalled Childhood Gender Identity/Gender Role Questionnaire: Psychometric Properties. *Sex Roles* 2006;54:469-483.
29. Jones BA, Bouman WP, Haycraft E, *et al.* The Gender Congruence and Life Satisfaction Scale (GCLS): Development and validation of a scale to measure outcomes from transgender health services. *Int J Transgend* 2018;20:63-80.
30. American Psychiatric Association. Available at: https://www.psychiatry.org/File%20Library/Psychiatrists/Practice/DSM/APA_DSM5_Level-1-Measure-Adult.pdf
31. Eriksson JM, Andersen LM, Bejerot S. RAADS-14 Screen: validity of a screening tool for autism spectrum disorder in an adult psychiatric population. *Mol Autism* 2013;4:49.
32. Woodbury-Smith MR, Robinson J, Wheelwright S, *et al.* Screening adults for Asperger Syndrome using the AQ: a preliminary study of its diagnostic validity in clinical practice. *J Autism Dev Disord* 2005;35:331-335.
33. Kessler RC, Adler L, Ames M, *et al.* The World Health Organization Adult ADHD Self-Report Scale (ASRS): a short screening scale for use in the general population. *Psychol Med* 2005;35:245-256.
34. Ward MF, Wender PH, Reimherr FW. The Wender Utah Rating Scale: an aid in the retrospective diagnosis of childhood attention deficit hyperactivity disorder. *Am J Psychiatry* 1993;150:885-890.
35. Bergman H, Källmén H. Alcohol use among Swedes and a psychometric evaluation of the alcohol use disorders identification test. *Alcohol Alcohol* 2002;37:245-251.
36. Berman AH, Bergman H, Palmstierna T, *et al.* Evaluation of the Drug Use Disorders Identification Test (DUDIT) in criminal justice and detoxification settings and in a Swedish population sample. *Eur Addict Res* 2005;11:22-31.
37. Lemmens JS, Valkenburg PM, Jochen P. Development and validation of a game addiction scale for adolescents. *Media psychology* 2009;12:77-95.
38. Volberg RA, Munck IM, Petry NM. A quick and simple screening method for pathological and problem gamblers in addiction programs and practices. *Am J Addict* 2011;20:220-227.
39. Berg KC, Peterson CB, Frazier P, *et al*. Psychometric evaluation of the eating disorder examination and eating disorder examination-questionnaire: a systematic review of the literature. *Int J Eat Disord* 2012;45:428-438.
40. Gustavsson JP, Bergman H, Edman G, *et al.* Swedish universities Scales of Personality (SSP): construction, internal consistency and normative data. *Acta Psychiatr Scand* 2000;102:217-225.
41. Sheehan DV, Harnett-Sheehan K, Raj BA. The measurement of disability. *Int Clin Psychopharmacol* 1996;11:89-95.
42. Ustün TB, Chatterji S, Kostanjsek N, *et al.* Developing the World Health Organization Disability Assessment Schedule 2.0. *Bull World Health Organ* 2010;88:815-823.

Table 1: Outline of the validated questionnaires used during different time points

Variables of interest	Questionnaires	M1	M3	M6	M12	M18	M19	M24	M25	M36	M37
GD symptoms	TCS[26]	✓	✓	✓	✓	✓		✓		✓	
Body satisfaction	BIS[27]	✓				✓				✓	
Gender identity and roles during childhood	RCGI[28]	✓									
Life satisfaction	GCLS[29]	✓		✓	✓	✓		✓		✓	
Mental health symptoms	DSM-5 screening[30]	✓	✓	✓	✓			✓			✓
Autistic features	RAADS-14[31]<br>AQ[32]	✓									
ADHD characteristics	ASRS[33]<br>WURS[34]	✓									
Alcohol use	AUDIT-C[35]	✓					✓				✓
Substance use	DUDIT-C[36]	✓					✓				✓
Gaming addiction	GAS[37]	✓					✓				✓
Gambling screening	NODS-CLiP[38]	✓					✓				✓
Eating disorder symptoms	EDE-Q[39]	✓					✓				✓
Personality traits	SSP[40]	✓							✓		
Functional impairment and disability	SDS[41]<br>WHODAS 2.0[42]	✓					✓				✓

M, month; ADHD; attention deficit hyperactivity disorder
TCS, Transgender Congruence Scale; BIS, Body Image Scale; RCGI, The Recalled Childhood Gender Identity / Gender Role Questionnaire; GCLS, The Gender Congruence and Life Satisfaction Scale; DSM-5 screening, Diagnostic and Statistical Manual 5th edition Self-Rated Level 1 Cross-Cutting Symptom Measure; RAADS-14, Ritvo Autism and Asperger Diagnostic Scale - 14; AQ, Autism Spectrum Quotient; ASRS, Adult ADHD self-report scale; WURS, Wender Utah Rating Scale; AUDIT-C, Alcohol Use Disorders Identification Test - Consumption; DUDIT-C, Drug Use Disorders Identification Test - Consumption; GAS, Game Addiction Scale; NODS-CLiP, Loss of Control, Lying, and Preoccupation Scale; EDE-Q, Eating Disorder Examination Questionnaire; SSP, Swedish Universities Scales of Personality; SDS, Sheehan Disability Scale; WHODAS 2.0, World Health Organization Disability Assessment Schedule 2.0

Table 2: Outline of the SKDS-specific questionnaires used during different time points

**Category**		**Survey I** Month 1	**Survey II** Month 3, 6, 12, 18, 24	**Survey III** Month 36
Background information	Age	✓		
	Birth assigned gender	✓	✓	✓
	Education	✓		✓
	Family	✓		✓
	Occupational status	✓		✓
	Marital/relationship status	✓		✓
	Current contact with gender identity clinic	✓	✓	✓
Gender identity	Gender identity	✓	✓	✓
	Preferred pronoun(s)	✓	✓	✓
	Openness about gender identity	✓	✓	✓
Gender dysphoria	Perceived distress from GD	✓	✓	✓
	Body satisfaction	✓	✓	✓
	Preferences regarding GAT	✓		✓
Gender affirming treatments (GAT)	Hormonal treatments	✓	✓	✓
	Top surgeries	✓	✓	✓
	Bottom surgeries	✓	✓	✓

For peer review only - http://bmjopen.bmj.com/site/about/guidelines.xhtml

	Speech therapy	✓	✓	✓
	Fertility preservation	✓	✓	✓
	Satisfaction with each of the treatments			✓
	Overall satisfaction with GAT			✓
Minority stress and support	Experiences of discrimination and violence	✓		
	Perceived social support	✓		
Sexuality	Sexual identity	✓		✓
	Sexual practice	✓		✓
	Satisfaction	✓		✓
Mental health	Mental health history	✓		
	Current psychiatric diagnoses	✓		✓
	Experiences of trauma	✓		✓
	Self-harm	✓		✓
	Suicidality	✓		✓
	Perceived overall mental health status	✓	✓	✓
Physical health	Chronic illness history	✓		✓

For peer review only

BMJ Open

Exploring gender dysphoria and related outcomes in a prospective cohort study: protocol for the Swedish Gender Dysphoria Study (SKDS)

Journal:	*BMJ Open*
Manuscript ID	bmjopen-2022-066571.R1
Article Type:	Protocol
Date Submitted by the Author:	23-Feb-2023
Complete List of Authors:	Özel, Fatih; Uppsala University, Department of Organismal Biology; Uppsala University, Centre for Women's Mental Health during the Reproductive Lifespan – WOMHER<br>Indremo, Malin; Uppsala University, Department of Medical Sciences, Psychiatry<br>Karamanis, Georgios; Uppsala University, Department of Medical Sciences, Psychiatry<br>Elofsson, Ulf; Uppsala University, Department of Medical Sciences, Psychiatry; Uppsala University, Department of Women's and Children's Health<br>Beckman, Ulrika; Sahlgrenska University Hospital, Department of Psychiatry<br>Fazekas, Attila; Lund University, Department of Psychiatry<br>Frisén, Louise; Karolinska Institute, Department of Clinical Neuroscience<br>Isaksson, Magnus; Uppsala University, Department of Medical Sciences<br>Sandström, Lotta; Karolinska University Hospital, ANOVA; Umeå Universitet, Department of Clinical Sciences, Psychiatry<br>Thelin, Nils; Linköping University Hospital, Division of Psychiatry<br>Tivesten, Åsa; University of Gothenburg, Wallenberg Laboratory for Cardiovascular and Metabolic Research, Department of Molecular and Clinical Medicine, Institute of Medicine, Sahlgrenska Academy; Sahlgrenska University Hospital, Department of Endocrinology<br>Wahlberg, Jeanette; Örebro University, Faculty of Medical Sciences<br>Skalkidou, Alkistis; Uppsala University, Department of Women's and Children's Health<br>Bodlund, Owe; Umeå Universitet, Department of Clinical Sciences, Psychiatry<br>Papadopoulos, Fotios C.; Uppsala University, Department of Medical Sciences, Psychiatry
**Primary Subject Heading**:	Epidemiology
Secondary Subject Heading:	Mental health, Epidemiology
Keywords:	Child & adolescent psychiatry < PSYCHIATRY, Adult psychiatry < PSYCHIATRY, EPIDEMIOLOGY

SCHOLARONE™
Manuscripts

Exploring gender dysphoria and related outcomes in a prospective cohort study: protocol for the Swedish Gender Dysphoria Study (SKDS)

Fatih Özel[1,2], Malin Indremo[3], Georgios Karamanis[3], Ulf Elofsson[3,4], Ulrika Beckman[5], Attila Fazekas[6], Louise Frisen[7], Magnus Isaksson[3], Lotta Sandström[8,9], Nils Thelin[10], Åsa Tivesten[11,12], Jeanette Wahlberg[13], Alkistis Skalkidou[4], Owe Bodlund[9], Fotios C Papadopoulos[3]

[1]Department of Organismal Biology, Uppsala University, Uppsala, Sweden
[2]Centre for Women's Mental Health during the Reproductive Lifespan – WOMHER, Uppsala University, Uppsala, Sweden
[3]Department of Medical Sciences, Uppsala University, Uppsala, Sweden
[4]Department of Women's and Children's Health, Uppsala University, Uppsala, Sweden
[5]Department of Psychiatry, Sahlgrenska University Hospital, Region Västra Götaland, Gothenburg, Sweden
[6]Department of Psychiatry, Lund University, Lund, Sweden
[7]Department of Clinical Neuroscience, Karolinska Institutet, Stockholm, Sweden
[8]ANOVA, Karolinska University Hospital, Stockholm, Sweden
[9]Department of Clinical Sciences/Psychiatry, Umeå University, Umeå, Sweden
[10]Division of Psychiatry, Linköping University Hospital, Linköping, Sweden
[11]Wallenberg Laboratory for Cardiovascular and Metabolic Research, Department of Molecular and Clinical Medicine, Institute of Medicine, Sahlgrenska Academy, University of Gothenburg, Gothenburg, Sweden
[12]Department of Endocrinology, Sahlgrenska University Hospital, Region Västra Götaland, Gothenburg, Sweden
[13]Faculty of Medical Sciences, Örebro University, Örebro, Sweden

Correspondence to:
Fotios C Papadopoulos
fotis.papadopoulos@neuro.uu.se

For peer review only

Abstract

Introduction

There has been a drastic increase in the reported number of people seeking for gender dysphoria in many countries over the last two decades. Yet, our knowledge of gender dysphoria and related outcomes is restricted due to the lack of high-quality studies employing comprehensive approaches. This longitudinal study aims to enhance our knowledge of gender dysphoria; different aspects will be scrutinized, focusing primarily on the psychosocial and mental health outcomes, prognostic markers, and secondarily on the underlying mechanisms for its origin.

Methods and analysis

The Swedish Gender Dysphoria Study is an ongoing multicenter longitudinal cohort study with 501 registered participants with gender dysphoria who are 15 years old or older. Participants at different phases of their clinical evaluation process can enter the study, and the expected follow-up duration is 3 years. The study also includes a comparison group of 458 age and county matched individuals without gender dysphoria. Data on the core outcomes of the study, which are gender incongruence and experienced gender dysphoria, body satisfaction and satisfaction with gender-affirming treatments, as well as other relevant outcomes including mental health, social functioning and life satisfaction are collected via web surveys. Two different research visits, before and after starting on gender-affirming hormonal treatment (if applicable), are planned to collect respective biological and cognitive measures. Data analysis will be performed using appropriate biostatistical methods. A power analysis showed that the current sample size is big enough to analyze continuous and categorical outcomes, and participant recruitment will continue until December 2022.

Ethics and dissemination

The ethical permission for this study was obtained from the Local Ethical Review Board in Uppsala, Sweden. Results of the study will be presented at national and international conferences and published in peer-reviewed journals. Dissemination will also be implemented through the Swedish Gender Dysphoria Study network in Sweden.

Strengths and limitations of this study

- The longitudinal design with a large sample size is the biggest strength of this multicenter cohort study, considering that gender dysphoria is an uncommon diagnosis.
- Having a comparison group consisting of individuals without gender dysphoria stands as a valuable advantage.
- A comprehensive assessment of psychosocial determinants is being performed.
- Participants enter the study at different stages of their clinical evaluation process, which partly results in missing baseline information.
- The representativeness of the target population could be affected by the fact that persons with more mental health issues might tend to participate less often or dropout in higher extent.

For peer review only

Introduction

Gender dysphoria (GD) is, according to the latest version of the Diagnostic and Statistical Manual of Mental Disorders (DSM-5),[1] a condition that involves distress due to incongruence between an individual's birth-assigned sex and gender identity. Persons with GD can identify themselves within binary and non-binary gender identities; non-binary identities may refer to having different gender identities simultaneously, having a neutral or fluid gender identity, not having a gender identity precisely and so forth. [2] During the last decades, there has been an increasing number of individuals with GD seeking gender-affirming health care in many countries.[3 4] This increase is more pronounced among the younger age groups, and is more noticeable among individuals who are assigned female at birth (AFAB).[5] Similar rising patterns for GD have also been demonstrated in Sweden recently. An increase in the incidence of GD was observed during the period 2004-2015 in Sweden, with the most profound increase among AFAB in the age group 10-17 (approximately 20-30% average yearly increase), while 10-17 year old assigned male at birth (AMAB) and both AFAB and AMAB in the age group 18-30 years had an average yearly increase of approximately 10-20%.[6] In the latest report of the National Board of Health and Welfare, 0.06% of the Swedish population was stated to have a GD diagnosis in 2018.[7] Even though an increasing trend in GD and the consequent need for gender-affirming health care is widely recognized, our understanding of GD and gender-affirming health care is substantially limited in many aspects.

The origin of GD is unknown but assumably multifactorial, with biological factors interacting with the psychosocial environment. There are established sex differences in the brain, concerning neuroanatomy, neurochemistry, as well as in neurocognition.[8] One hypothesis for the emergence of GD is that the processes of sexual differentiation of the genitals and the brain may be incongruent.[8] However, evidence from multiple neuroimaging studies has not given support to this, and alternative hypotheses have been proposed. [9 10] Genetic factors have also been observed to play a role in GD, and twin studies have been mostly employed to enlighten the development in GD. [11-13] Even though the existing evidence remains largely inconclusive, genetic factors seem to be involved through altered sex hormone signaling; the estrogen and androgen receptors seem to be implicated in the genetic basis of GD.[14] Moreover, epigenetic alterations among individuals with GD have been recently proposed,[15] supporting evidence highlighting the importance of the interaction between genetic architecture and environment in the development of GD. Thus, early-life environmental exposures could potentially be associated with the biological underpinning of GD via epigenetic modifications. Yet, the current evidence on biological determinants of GD are inconclusive in the literature.

An important aspect in the field of GD research relates to the mental health of individuals with GD. A growing body of evidence shows that persons with GD are more vulnerable to mental illnesses. A recent study utilizing Swedish population register data has reported that mood and anxiety disorders, use of antidepressant or anxiolytic medication and hospitalization rates due to suicide attempts are more

For peer review only - http://bmjopen.bmj.com/site/about/guidelines.xhtml

common among persons with GD in comparison to the general population.[16] In addition to psychiatric diagnoses, transgender persons' quality of life is radically affected, with reported lower life satisfaction.[17] The aforementioned adverse consequences on the mental well-being of individuals with GD could be explained through experienced dysphoria and stigma-related stress. This framework of stigmatization and discrimination in this context is conventionally called minority stress and was established almost 20 years ago.[18] According to the minority stress model, gender minority groups endure particular stressors such as discrimination, lack of social support and violence, and this intense exposure to stressors could result in mental health issues. Individuals with GD are unfortunately no exception to minority gender groups and their health is influenced by different sources of stigma-related stress.[17 19] More research is required on the experience of discrimination and violence by persons with GD and its effects on their mental health.

Gender-affirming medical treatment is an additional dimension that needs to be addressed in research. Treatment guidelines are provided in the World Professional Association for Transgender Health's (WPATH) Standards of Care, currently in its 8th version.[20] Depending on individual needs and wishes, treatments involve gender-affirming hormonal treatment (GAHT), gender-affirming surgical procedures, consultations and potential interventions for reproductive health, voice therapy, hair removal and use of prostheses. GAHT consists mainly of estrogens and antiandrogens for AMAB and testosterone for AFAB. The goal of GAHT is to induce the development of bodily characteristics congruent with the self-identified gender, thereby improving mental health and quality of life. For children and adolescents, puberty suppression with GnRH analogues is implemented to delay the development of secondary sexual characteristics. The intention is to alleviate the distress associated with the development of secondary sex characteristics, thereby providing a time for ongoing discussion and exploration of gender identity before deciding whether to take more irreversible steps. Previous studies posit that GAHT ameliorates the symptoms of GD, increases the quality of life, alleviates depression, anxiety, suicidality and improves psychosocial functioning among both young and adult individuals.[21-24] One should still acknowledge the limitations of interpreting the results from previous research. Moreover, the reports on the potential adverse health outcomes related to GAHT make research in this dimension even more indispensable.[25 26]

There has been an increase in research studies related to GD in parallel to the increase in GD incidence. Still, we lack evidence focused on some particular aspects of GD; such as biological and psychological determinants, mental health and psychosocial outcomes and the impact of gender-affirming treatment (GAT) on these outcomes. The majority of the studies conducted in the field of GD have methodological limitations, with small sample sizes, cross-sectional design and no comparison groups. Larger longitudinal studies are required to improve our understanding of GD and associated outcomes. More research is especially warranted among adolescents and young adults with GD, as those are the age groups with the most profound incidence increase during the last decade.

For peer review only - http://bmjopen.bmj.com/site/about/guidelines.xhtml

The Swedish Gender Dysphoria Study (Svenska Könsdysforistudien - SKDS) thus primarily aims to investigate (1) mental health outcomes associated with GD, for instance, depressive and anxiety symptoms and suicidality; (2) psychosocial outcomes in relation to GD, such as quality of life, global functioning and social vulnerability (along with support, discrimination and violence); (3) different aspects of GAT, including the impact on mental health and psychosocial outcomes; and secondarily (4) biological correlates of GD containing the effects of early-life environmental exposures, genetic underpinnings, epigenetic alterations and potential prognostic biological markers.

Methods and analysis

Study design and participants

SKDS (www.skds.se) is an ongoing multicenter longitudinal cohort study in Sweden, which was established in 2016. All participants in contact with one of the associated gender dysphoria evaluation clinics are eligible to be asked to participate. Having contact with a clinic for gender dysphoria, being at least 15 years old and speaking Swedish were set as the inclusion criteria. Relevant information about the study is sent to all participants and written informed consent is obtained. The right of leaving the study without any obligation to report a reason for that and the fact that participating or not in the study has no effect on the received healthcare is clearly indicated during the informed consent process. Separate informed consents are asked for different parts of the study (e.g. participating in surveys, research visits, online cognitive tests, giving biological samples, etc). Recruitment of participants is ongoing in the GD clinics in Alingsås/Gothenburg, Linköping, Lund/Malmö, Stockholm (center for evaluation of children), Umeå, and Uppsala. In Sweden, the evaluation process and treatment for GD is centralized to these six regional clinics working in multidisciplinary teams, all following a national consensus program.

The participants of SKDS are followed up longitudinally from inclusion to three years later, with several assessments of variables of interest. Due to the naturalistic design of the study, the participants can be at different phases in their clinical evaluation process when they enter the study. The recruitment of the participants is expected to be finished in December 2022; therefore, the follow-up is predicted to be finalized in December 2025.

There are currently 501 registered participants with GD in SKDS. In January 2019, the governmental agency Statistics Sweden provided 30 random controls with no registered diagnosis of GD up to that time (15 with male legal gender and 15 with female legal gender) for each participant in the study. To recruit controls, an invitation letter and one reminder were sent via mail to 8790 persons in total; a comparison group thus was constituted with 458 age and county matched controls without a diagnosis of GD. The controls are only assessed once with the same baseline instruments as the participants with GD. Regarding the low participation rate for the controls, the dropout analysis conducted by Statistics Sweden showed higher dropout rates for controls with male legal gender, those born outside Sweden and those without Swedish citizenship. No other major biases were observed for age, income or municipality. The second round of recruitment of controls will be executed at the beginning of 2023, in order to acquire more controls for recruited participants since 2019.

For peer review only - http://bmjopen.bmj.com/site/about/guidelines.xhtml

In order to plan and follow the study coordination and progress as well as the utilization of all collected data, a national steering committee for the SKDS was created in November 2015, consisting of clinicians and researchers in the field of psychiatry and endocrinology from the collaborating clinics. Representatives from interest organizations, including Transammans and RFSL Ungdom in Stockholm and Uppsala, have an advisory role in the SKDS.

Data collection and measures

1. Main outcomes and psychosocial measures

Longitudinal web surveys are the main tools for data collection in the current study. Sociodemographic characteristics and outcomes of relevance for GD are measured through previously validated instruments listed in Table 1, as well as three specific surveys for SKDS, Surveys I-III, described in Table 2. The core outcomes of the current study are gender incongruence and experienced gender dysphoria, body satisfaction, and satisfaction with GAT. Assessment of the satisfaction with each of the received GAT, as well as overall satisfaction with GAT, is covered in depth in the last SKDS-specific survey. For each of the received treatments, the participants are asked the following questions: “how satisfied are you with the physical result of treatment x?”, ”how has treatment x affected your mental well-being?” and “if you were to decide on initiating treatment x today, would you make the same decision again?”.

In addition to the core outcomes, the included instruments cover general mental health status, previous and current psychiatric diagnoses, traits of autism spectrum disorder and attention deficit hyperactivity disorder, alcohol and substance use, social functioning and life satisfaction. Furthermore, a scale on potential side effects of treatment is administered after the initiation of GAT.

2. Cognitive measures

All participants who have not started on gender-affirming hormonal treatment are invited to undertake a computer-based neurocognitive assessment battery, called WebNeuro, which can be administered online or with a local installation.[27] Different domains are addressed within the battery including sensorimotor, memory, executive functioning, attention, and social cognition. Somatomaps, an online tool for assessing body perception, is also administered to all participants.[28]

Patients recruited from the gender dysphoria evaluation clinic in Uppsala are also invited to a research visit at the Uppsala University Hospital where they are asked to undertake specific neurocognitive tests called Cambridge Gambling Task (CGT) and Stockings of Cambridge (SOC) from the CANTAB battery.[29] All cognitive tests are performed at baseline, before GAHT and at least six months after start of GAHT.

3. Biological measures

For genetic and epigenetic analyses, all participants are asked to contribute with saliva samples, deciduous teeth and the consent to use newborn dried blood spots which are stored in the national PKU-biobank. Saliva samples will also be collected after a period of at least six months after starting

For peer review only - http://bmjopen.bmj.com/site/about/guidelines.xhtml

on GAHT for individuals without GAHT at baseline in order to do epigenetic analyses related to the GAHT.

Participants recruited from the gender dysphoria evaluation clinic in Uppsala are also invited to take part in a research visit, contributing to more biological measures before and after the initiation of GAHT. The measures consist of blood samples to analyze hormonal levels and possible changes in the immune system, a physical examination including blood pressure, weight and hip-waist ratio, as well as measuring bioimpedance and heart rate variability. The first research visit is conducted during baseline assessment and a follow-up visit at least six months after initiation of GAHT.

These biological samples will be used in order to study potential biological correlates of GD consisting of the effects of early-life environmental exposures, genetic underpinnings, epigenetic alterations as well as potential prognostic biomarkers.

4. Electronic Health Records (EHR) and national register data

Information related to participants' gender dysphoria evaluation can be retrieved from the EHR for those who have given their consent. Similarly, for those with consent, a linkage will be made to the Swedish national registers, such as National Patient Register at the National Board of Health and Welfare and Statistics Sweden. These two information sources will be used to supplement the information collected with the web surveys.

Statistical Analysis

The research team of SKDS will perform the statistical analyses using the most recent version of the statistical software R. We will manage different types of missing data with different approaches. First, a potential bias that could arise from loss to follow-up will be evaluated; characteristics of missing participants will be addressed with dropout analysis and presented. Second, missing data and loss to follow-up will be diminished with chart review of clinical records and linkage to national health registers. We will also execute multiple imputation where it is required, and pooled results will be presented from imputed datasets.

Initially, summary statistics including the descriptive characteristics of participants will be provided. Cross-sectional and longitudinal relationships will be assessed using the distinctive outcomes we mentioned above. Logistic regression analysis will be used for the categorical outcomes, such as having been diagnosed with GD or not. Continuous outcomes will be analyzed with linear regression analysis. For longitudinal relations, we will conduct linear mixed models with repeated measures. We will use directed acyclic graphs (DAG) to identify confounders and mediators. Confounders will be adjusted in different models, whereas potential mediators will also be assessed with mediation analysis. We will apply multiple testing correction wherever necessary.

Regarding statistical power analysis, we need data for 52 participants of each birth-assigned sex in order to detect a 25% increase from a mean Transgender Congruence Scale (TCS) score of 2.1, with alpha=0.05 and power=90%, using a Mann-Whitney U test for the outcome of GD. Concerning the

For peer review only - http://bmjopen.bmj.com/site/about/guidelines.xhtml

categorical mental health outcomes, expecting a change from 30% to 15% (as we have seen for the prevalence of self-harm thoughts in preliminary analysis from the SKDS-data), we need 161 participants for alpha=0.05 and power=90%, using a chi-squared test. For a smaller change from 30% to 20%, we need 294 participants for alpha=0.05 and power=80%. The participation rate for SKDS is approximately 70%. Among those participants, approximately 70% actively participate in the majority of our surveys. The expected final number of recruited participants in the study is around 600, which leaves nearly 400 actively engaged participants.

Patient and public involvement

The study was initially designed by the research group comprising of clinicians and researchers working clinically with patients with GD. During the recruitment phase a collaboration was established with representatives from interest organizations in Sweden, which are RFSL Ungdom and Transammans; this collaboration is ongoing with regular meetings. Persons with GD were involved in the design of SKDS through interest organization representatives; the researchers were informed by the representatives concerning the contemporary needs of individuals with GD, and the research questions, methods and outcome measures were adjusted and further developed based on the feedback received. Interest organizations will be asked to contribute to the dissemination of the results through their networks. The results will also be disseminated through the project's website which is publicly accessible.

Ethics and dissemination

Ethical awareness has critical importance in clinical studies since patients might feel pressured to participate to access health care services. In SKDS, the voluntary participation and the fact that participation has no effect whatsoever on received care are clarified both orally and in writing. Study participants were also informed that they could cancel their participation without any justification at any stage of follow-up. Furthermore, data breach might become an important risk, as sensitive information at the individual level is stored. However, we have taken all measures, such as pseudonymization, storage of the data on secure servers at Uppsala University and separating the personal identifiers (i.e. name and personal identity number) from the self-reports and other collected data. The study was approved by the Ethical Review Board in Uppsala (Dnr:2016/013).

We plan to disseminate the research findings from the SKDS through publication in peer-reviewed journals and presentation at relevant national and international conferences, as well as at dedicated seminars or workshops organized by the involved researchers, which could be also of interest to governmental and non-governmental bodies. Dissemination will be assured through the project's website (www.skds.se), as well as social media platforms. The results will be delivered to all clinical teams working with GD evaluation in Sweden through direct communication within the SKDS network. Contact will also be initiated with the national societies of general physicians, psychiatrists, psychologists, endocrinologists, as well as obstetricians and gynaecologists in Sweden.

Authors' contributions

For peer review only - http://bmjopen.bmj.com/site/about/guidelines.xhtml

AF, AS, JW, LF, LS, MIs, NT, OB, UB, ÅT and FCP were responsible for the conception and design of the study. FÖ drafted the protocol with significant contributions by MIn, GK, UE and FCP. All authors have critically revised the draft for intellectual content and approved the final manuscript. FCP is the principal investigator of the study.

Funding statement

This work is supported by Region Uppsala, the Foundation of Nicke and Märta Nasvell, FORTE (dnr: 2021-01968) and Centre for Women's Mental Health during the Reproductive Lifespan – WOMHER.

Competing interests statement

None declared.

Acknowledgments

We would like to express our gratitude to all the study participants and the clinicians at the collaborating clinics. Moreover, we acknowledge Lucas Blixt from RFSL Ungdom Uppsala, Tom Summanen from Transammans and Vierge Hård from RFSL Ungdom for their advisory roles in the study.

References

1. American Psychiatric Association. Diagnostic and Statistical Manual of Mental Disorder 5th edition. *American Psychiatric Pub* 2013.
2. Richards C, Bouman WP, Barker M. Non-binary genders. *London: Pal grave Macmillan* 2017.
3. Arcelus J, Bouman WP, Van Den Noortgate W, *et al*. Systematic review and meta-analysis of prevalence studies in transsexualism. *Eur Psychiatry* 2015;30:807-815.
4. de Graaf NM, Carmichael P, Steensma TD, *et al*. Evidence for a Change in the Sex Ratio of Children Referred for Gender Dysphoria: Data From the Gender Identity Development Service in London (2000-2017). *J Sex Med* 2018;15:1381-1383.
5. Aitken M, Steensma TD, Blanchard R, *et al*. Evidence for an altered sex ratio in clinic-referred adolescents with gender dysphoria. *J Sex Med* 2015;12:756-763.
6. Indremo M, White R, Frisell T, *et al*. Validity of the Gender Dysphoria diagnosis and incidence trends in Sweden: a nationwide register study. *Sci Rep* 2021;11:16168.
7. Socialstyrelsen, Utvecklingen av diagnosen könsdysfori, 2020. Available: https://www.socialstyrelsen.se/globalassets/sharepoint-dokument/artikelkatalog/ovrigt/2020-2-6600.pdf
8. Smith ES, Junger J, Derntl B, *et al*. The transsexual brain--A review of findings on the neural basis of transsexualism. *Neurosci Biobehav Rev* 2015;59:251-266.
9. Manzouri A, Savic I. Possible Neurobiological Underpinnings of Homosexuality and Gender Dysphoria. *Cereb Cortex* 2019;29:2084-2101.
10. Majid DSA, Burke SM, Manzouri A, et al. Neural Systems for Own-body Processing Align with Gender Identity Rather Than Birth-assigned Sex. *Cereb Cortex* 2020;30:2897-2909.
11. Diamond M. Transsexuality among twins: identity concordance, transition, rearing, and orientation. *IntJ Transgend*, 2013;*14*(1):24-38.
12. Sasaki S, Ozaki K, Yamagata S, *et al.* Genetic and Environmental Influences on Traits of Gender Identity Disorder: A Study of Japanese Twins Across Developmental Stages. *Arch Sex Behav*. 2016;45(7):1681-1695.
13. Karamanis G, Karalexi M, White R, *et al.* Gender dysphoria in twins: a register-based population study. *Sci Rep*. 2022;12(1):13439.
14. Foreman M, Hare L, York K, *et al.* Genetic Link Between Gender Dysphoria and Sex Hormone Signaling. *J Clin Endocrinol Metab* 2019;104(2):390-396.
15. Ramirez K, Fernández R, Collet S, *et al.* Epigenetics Is Implicated in the Basis of Gender Incongruence: An Epigenome-Wide Association Analysis. *Front Neurosci* 2021;15:701017.

For peer review only - http://bmjopen.bmj.com/site/about/guidelines.xhtml

16. Bränström R, Pachankis JE. Reduction in Mental Health Treatment Utilization Among Transgender Individuals After Gender-Affirming Surgeries: A Total Population Study. *Am J Psychiatry* 2020;177:727-734.
17. Bränström R, Pachankis JE. Country-level structural stigma, identity concealment, and day-to-day discrimination as determinants of transgender people's life satisfaction. *Soc Psychiatry Psychiatr Epidemiol* 2021;56:1537-1545.
18. Meyer IH. Prejudice, social stress, and mental health in lesbian, gay, and bisexual populations: conceptual issues and research evidence. *Psychol Bull* 2003;129:674-697.
19. White Hughto JM, Reisner SL, Pachankis JE. Transgender stigma and health: A critical review of stigma determinants, mechanisms, and interventions. *Soc Sci Med* 2015;147:222-231.
20. Coleman E, Radix AE, Bouman WP, *et al.* Standards of Care for the Health of Transgender and Gender Diverse People, Version 8. *IntJ Transgend*, 2022,*23*(Suppl 1):1–259.
21. Murad MH, Elamin MB, Garcia MZ, *et al*. Hormonal therapy and sex reassignment: a systematic review and meta-analysis of quality of life and psychosocial outcomes. *Clin Endocrinol (Oxf)* 2010;72:214-231.
22. Mahfouda S, Moore JK, Siafarikas A, *et al*. Puberty suppression in transgender children and adolescents. *Lancet Diabetes Endocrinol* 2017;5:816-826.
23. Chew D, Anderson J, Williams K, *et al*. Hormonal Treatment in Young People With Gender Dysphoria: A Systematic Review. *Pediatrics* 2018;141:e20173742.
24. Kuper LE, Stewart S, Preston S, *et al*. Body Dissatisfaction and Mental Health Outcomes of Youth on Gender-Affirming Hormone Therapy. *Pediatrics* 2020;145:e20193006.
25. Getahun D, Nash R, Flanders WD, *et al.* Cross-sex Hormones and Acute Cardiovascular Events in Transgender Persons: A Cohort Study. *Ann Intern Med* 2018;169:205-213.
26. Nota NM, Wiepjes CM, de Blok CJM, *et al.* Occurrence of Acute Cardiovascular Events in Transgender Individuals Receiving Hormone Therapy. *Circulation* 2019;139:1461-1462.
27. Silverstein SM, Berten S, Olson P, *et al.* Development and validation of a World-Wide-Web-based neurocognitive assessment battery: WebNeuro. *Behav Res Methods* 2007;39:940-949.
28. Ralph-Nearman C, Arevian AC, Puhl M, *et al.* A Novel Mobile Tool (Somatomap) to Assess Body Image Perception Pilot Tested With Fashion Models and Nonmodels: Cross-Sectional Study. *JMIR Ment Health* 2019;6:e14115.
29. Available: https://www.cambridgecognition.com/products/cognitive-research/
30. Iliadis SI, Axfors C, Friberg A, *et al.* Psychometric properties and concurrent validity of the Transgender Congruence Scale (TCS) in the Swedish setting. *Sci Rep* 2020;10:18701.
31. Lindgren TW, Pauly IB. A body image scale for evaluating transsexuals. *Arch Sex Behav* 1975;4:639-656.
32. Zucker K, Mitchell J, Bradley S, *et al.* The Recalled Childhood Gender Identity/Gender Role Questionnaire: Psychometric Properties. *Sex Roles* 2006;54:469-483.
33. Jones BA, Bouman WP, Haycraft E, *et al.* The Gender Congruence and Life Satisfaction Scale (GCLS): Development and validation of a scale to measure outcomes from transgender health services. *Int J Transgend* 2018;20:63-80.
34. American Psychiatric Association. Available at: https://www.psychiatry.org/File%20Library/Psychiatrists/Practice/DSM/APA_DSM5_Level-1-Measure-Adult.pdf
35. Eriksson JM, Andersen LM, Bejerot S. RAADS-14 Screen: validity of a screening tool for autism spectrum disorder in an adult psychiatric population. *Mol Autism* 2013;4:49.
36. Woodbury-Smith MR, Robinson J, Wheelwright S, *et al.* Screening adults for Asperger Syndrome using the AQ: a preliminary study of its diagnostic validity in clinical practice. *J Autism Dev Disord* 2005;35:331-335.
37. Kessler RC, Adler L, Ames M, *et al.* The World Health Organization Adult ADHD Self-Report Scale (ASRS): a short screening scale for use in the general population. *Psychol Med* 2005;35:245-256.
38. Ward MF, Wender PH, Reimherr FW. The Wender Utah Rating Scale: an aid in the retrospective diagnosis of childhood attention deficit hyperactivity disorder. *Am J Psychiatry* 1993;150:885-890.
39. Bergman H, Källmén H. Alcohol use among Swedes and a psychometric evaluation of the alcohol use disorders identification test. *Alcohol Alcohol* 2002;37:245-251.
40. Berman AH, Bergman H, Palmstierna T, *et al.* Evaluation of the Drug Use Disorders Identification Test (DUDIT) in criminal justice and detoxification settings and in a Swedish population sample. *Eur Addict Res* 2005;11:22-31.

For peer review only - http://bmjopen.bmj.com/site/about/guidelines.xhtml

41. Lemmens JS, Valkenburg PM, Jochen P. Development and validation of a game addiction scale for adolescents. *Media psychology* 2009;12:77-95.
42. Volberg RA, Munck IM, Petry NM. A quick and simple screening method for pathological and problem gamblers in addiction programs and practices. *Am J Addict* 2011;20:220-227.
43. Berg KC, Peterson CB, Frazier P, *et al*. Psychometric evaluation of the eating disorder examination and eating disorder examination-questionnaire: a systematic review of the literature. *Int J Eat Disord* 2012;45:428-438.
44. Gustavsson JP, Bergman H, Edman G, *et al.* Swedish universities Scales of Personality (SSP): construction, internal consistency and normative data. *Acta Psychiatr Scand* 2000;102:217-225.
45. Sheehan DV, Harnett-Sheehan K, Raj BA. The measurement of disability. *Int Clin Psychopharmacol* 1996;11:89-95.
46. Ustün TB, Chatterji S, Kostanjsek N, *et al.* Developing the World Health Organization Disability Assessment Schedule 2.0. *Bull World Health Organ* 2010;88:815-823.

For peer review only

For peer review only - http://bmjopen.bmj.com/site/about/guidelines.xhtml

Table 1: Outline of the validated questionnaires used during different time points

**Variables of interest**	**Questionnaires**	**M1**	**M3**	**M6**	**M12**	**M18 – M19**	**M24 – M25**	**M36 – M37**
GD symptoms	TCS[30]	✓	✓	✓	✓	✓	✓	✓
Body satisfaction	BIS[31]	✓				✓		✓
Gender identity and roles during childhood	RCGI[32]	✓						
Life satisfaction	GCLS[33]	✓		✓	✓	✓	✓	✓
Mental health symptoms	DSM-5 screening[34]	✓	✓	✓	✓		✓	✓
Autistic features	RAADS-14[35]<br>AQ[36]	✓						
ADHD characteristics	ASRS[37]<br>WURS[38]	✓						
Alcohol use	AUDIT-C[39]	✓				✓		✓
Substance use	DUDIT-C[40]	✓				✓		✓
Gaming addiction	GAS[41]	✓				✓		✓
Gambling screening	NODS-CLiP[42]	✓				✓		✓
Eating disorder symptoms	EDE-Q[43]	✓				✓		✓
Personality traits	SSP[44]	✓					✓	
Functional impairment and disability	SDS[45]<br>WHODAS 2.0[46]	✓				✓		✓

M, month; ADHD; attention deficit hyperactivity disorder

TCS, Transgender Congruence Scale; BIS, Body Image Scale; RCGI, The Recalled Childhood Gender Identity / Gender Role Questionnaire; GCLS, The Gender Congruence and Life Satisfaction Scale; DSM-5 screening, Diagnostic and Statistical Manual 5th edition Self-Rated Level 1 Cross-Cutting Symptom Measure; RAADS-14, Ritvo Autism and Asperger Diagnostic Scale - 14; AQ, Autism Spectrum Quotient; ASRS, Adult ADHD self-report scale; WURS, Wender Utah Rating Scale; AUDIT-C, Alcohol Use Disorders Identification Test - Consumption; DUDIT-C, Drug Use Disorders Identification Test - Consumption; GAS, Game Addiction Scale; NODS-CLiP, Loss of Control, Lying, and Preoccupation Scale; EDE-Q, Eating Disorder Examination Questionnaire; SSP, Swedish Universities Scales of Personality; SDS, Sheehan Disability Scale; WHODAS 2.0, World Health Organization Disability Assessment Schedule 2.0

For peer review only - http://bmjopen.bmj.com/site/about/guidelines.xhtml

Table 2: Outline of the SKDS-specific questionnaires used during different time points

**Category**		**Survey I** Month 1	**Survey II** Month 3, 6, 12, 18, 24	**Survey III** Month 36
Background information	Age	✓		
	Birth assigned gender	✓	✓	✓
	Education	✓		✓
	Family	✓		✓
	Occupational status	✓		✓
	Marital/relationship status	✓		✓
	Current contact with gender identity clinic	✓	✓	✓
Gender identity	Gender identity	✓	✓	✓
	Preferred pronoun(s)	✓	✓	✓
	Openness about gender identity	✓	✓	✓
Gender dysphoria	Perceived distress from GD	✓	✓	✓
	Body satisfaction	✓	✓	✓
	Preferences regarding GAT	✓		✓
Gender affirming treatments (GAT)	Hormonal treatments	✓	✓	✓
	Top surgeries	✓	✓	✓
	Bottom surgeries	✓	✓	✓

For peer review only - http://bmjopen.bmj.com/site/about/guidelines.xhtml

	Speech therapy	✓	✓	✓
	Fertility preservation	✓	✓	✓
	Satisfaction with each of the treatments			✓
	Overall satisfaction with GAT			✓
Minority stress and support	Experiences of discrimination and violence	✓		
	Perceived social support	✓		
Sexuality	Sexual identity	✓		✓
	Sexual practice	✓		✓
	Satisfaction	✓		✓
Mental health	Mental health history	✓		
	Current psychiatric diagnoses	✓		✓
	Experiences of trauma	✓		✓
	Self-harm	✓		✓
	Suicidality	✓		✓
	Perceived overall mental health status	✓	✓	✓
Physical health	Chronic illness history	✓		✓

For peer review only

For peer review only - http://bmjopen.bmj.com/site/about/guidelines.xhtml
